# Bilateral Facial Paralysis in an Atypical Clinical Onset of COVID-Associated Guillain-Barre Syndrome

**DOI:** 10.7759/cureus.63498

**Published:** 2024-06-30

**Authors:** Ahmad Taftaf, Claudine A Vera Cruz, Haytham Al-Qasmi, Mohammed Al-Qasmi

**Affiliations:** 1 Internal Medicine, Hurley Medical Center, Flint, USA; 2 Neurology, Michigan State University College of Human Medicine, Flint, USA; 3 Neurology, University of Michigan, Ann Arbor, USA; 4 Neurology, Hurley Medical Center, Flint, USA

**Keywords:** post-covid complications, neurological effects of coronavirus, bifacial weakness with paraesthesia, guillain barre syndrome (gbs), coronavirus disease (covid-19)

## Abstract

Coronavirus disease (COVID-19) has been associated with a diverse range of extrapulmonary manifestations since its global outbreak in 2019. One of its rare complications is Guillain-Barre Syndrome (GBS), a post-infectious neurological disorder that manifests with a characteristic ascending limb paralysis. Here, we describe the atypical case of a 42-year-old African American male who developed bilateral facial paralysis within five weeks of testing positive for COVID-19. Initial diagnostic imaging and blood studies were negative for acute pathology. Albuminocytological dissociation found in a subsequent analysis of the patient’s cerebrospinal fluid and his appropriate therapeutic response to intravenous immunoglobulin (IVIg) indicated GBS as the most likely diagnosis.

## Introduction

Severe acute respiratory syndrome coronavirus 2 (SARS-CoV-2) has been recognized as a potent respiratory pathogen since the global outbreak of coronavirus disease in 2019 (COVID-19) [1]. However, a diverse range of extrapulmonary manifestations has been identified in recent studies [2-4]. One complication is Guillain-Barre syndrome (GBS), a rare neurological disorder that manifests with a characteristic ascending limb paralysis preceded by an acute respiratory or gastrointestinal infection [5,6]. Bilateral paralysis of the facial nerves, particularly at the clinical onset of GBS, is extremely unusual. This variant, classified as bifacial weakness with paresthesias (BFP) by the GBS Classification Group [6], was reported as the primary presenting symptom in only 5% (4/73) of cases in a comprehensive study of COVID-associated GBS [2]. Here, we describe the atypical case of a 42-year-old African American male who developed bilateral facial paralysis within five weeks of testing positive for COVID-19.

## Case presentation

A 42-year-old African American male with no prior medical history presented to his primary care physician due to the persistence of fatigue, vomiting, and diarrhea four weeks after testing positive for COVID-19. It was recommended for the patient to proceed to the emergency department (ED) due to concerns for clinical dehydration. ED workup revealed elevated levels of creatinine and blood urea nitrogen at 7.0 mg/dL and 153 mg/dL, respectively, hyponatremia at 122 mEq/L, and a high anion gap of 18. An EKG showed a significantly prolonged QT interval of 600 ms, and urinalysis showed trace hematuria and 2+ proteinuria. An ultrasound revealing increased echogenicity of bilateral renal parenchyma was suggestive of chronic disease. The patient was subsequently admitted to the hospital for management of an acute kidney injury secondary to severe dehydration, and treatment with intravenous (IV) rehydration and electrolyte replacement was initiated. 

**Figure 1:**
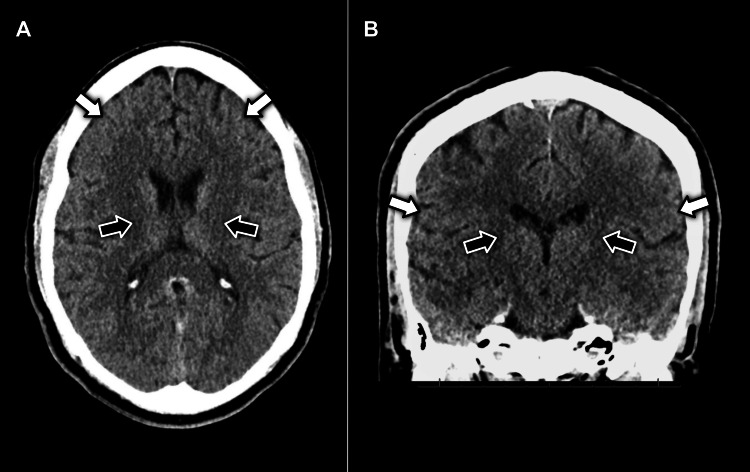
(A) axial and (B) coronal view CT imaging of the head without contrast displaying no evidence of pathology in the facial motor cortex (white arrows) or neuron pathways in the internal capsule (black arrows)

On day five of admission, the patient developed sudden-onset slurred speech, prompting activation of the hospital stroke protocol. On initial assessment, he was noted to be hemodynamically stable, alert and oriented, and in no acute distress. Neurological examination was significant for bilateral facial weakness and diminished deep tendon reflexes throughout. The slurred speech initially reported was found to be dysarthria due to motor impairment of the face, resulting in poor articulation. There were otherwise no changes to his baseline mental status, sensation, motor function, strength, or coordination, and a non-contrast CT scan of the head was negative for acute pathology (Figure 1).

**Figure 2:**
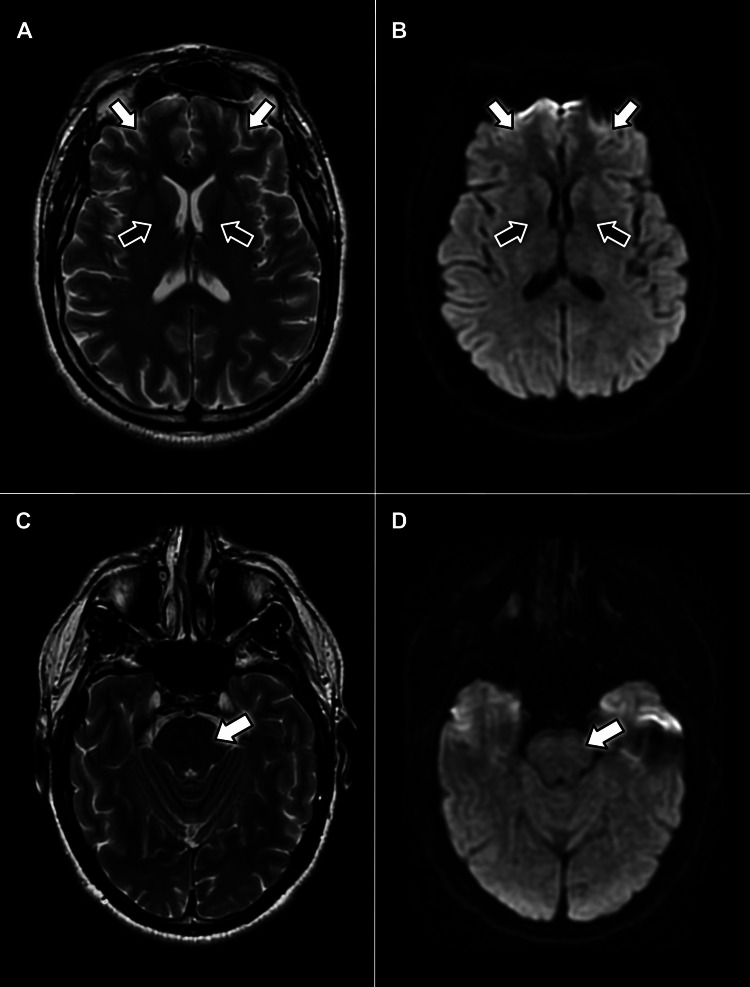
Axial MRI scans without contrast displaying no evidence of pathology in the facial motor cortex (white arrows) or neuron pathways in the internal capsule (black arrows) on T2-weighted (A) or diffusion-weighted (B) sequences. Additionally, no hyperintense signals appear within the pons on T2-weighted (C) or diffusion-weighted (D) sequences

Over the next few hours, the progression of facial nerve weakness led to worsening dysarthria and the development of dysphagia, with the patient displaying an inability to fully close the mouth, uncoordinated chewing, and difficulty propelling food towards the oropharynx. Given the patient's ongoing renal insufficiency, encephalopathy and central pontine myelinolysis were considered; however, serial lab studies (Table 1) and a non-contrast brain MRI without evidence of central pathology (Figure 2) deemed these conditions less likely. A comprehensive workup for metabolic, autoimmune, and infectious etiologies was completed, all of which returned negative results (Table 2). Consequently, cerebrospinal fluid (CSF) was collected via a spinal tap for analysis (Table 3). CSF profiling revealed red and white blood cell counts of 0, a normal glucose level of 47 mg/dL, and a mildly elevated protein level of 48 mg/dL. Based on the albuminocytologic dissociation observed in the CSF, COVID-associated GBS was indicated as the most likely diagnosis. The patient received a five-day course of intravenous immunoglobulin (IVIg) and experienced symptomatic regression throughout the admission. Upon discharge, he was advised to establish care with neurology on an outpatient basis for an electromyographic (EMG) study and further management, but was ultimately lost to follow-up.

**Table 1:** Serial basic metabolic panel BUN - blood urea nitrogen; H - high; L - low

Component, reference range, and units	Day 1 (admission)	Day 2	Day 3	Day 4	Day 5 (code stroke)	Day 6	Day 11 (discharge)
Sodium (136-145 mEq/L)	122 (L)	126 (L)	126 (L)	130 (L)	134 (L)	136	136
Potassium (3.4-5.1 mEq/L)	4.2	3.6	3.4	3.7	3.8	3.9	4.0
Chloride (98-107 mEq/L)	88 (L)	95 (L)	98	103	109 (H)	109 (H)	104
CO_2 _(23-29 mEq/L)	16 (L)	18 (L)	17 (L)	18 (L)	18 (L)	20 (L)	24
BUN (6-20 mg/dL)	153 (H)	139 (H)	132 (H)	105 (H)	83 (H)	62 (H)	15
Creatinine (0.5-1.1 mg/dL)	7 (H)	6 (H)	5.6 (H)	4.9 (H)	4.3 (H)	3.6 (H)	2.0 (H)
Glucose (70-100 mg/dL)	111 (H)	96	85	94	135 (H)	91	86
Calcium (8.7-10.4 mg/dL)	8.4 (L)	7.1 (L)	7.3 (L)	7.4 (L)	7.6 (L)	8.2 (L)	8 (L)
Anion gap (3-14 mEq/L)	18 (H)	13	11	9	7	7	8
Estimated glomerular filtration rate (>60mL/min/1.73m^2^)	10 (L)	12 (L)	13 (L)	16 (L)	18 (L)	23 (L)	46 (L)

**Table 2:** Diagnostic Bloodwork C-ANCA - cytoplasmic antineutrophil cytoplasmic antibodies; P-ANCA - perinuclear anti-neutrophil cytoplasmic antibodies; SSA - Sjögren's-syndrome-related antigen A

Component	Value	Reference range & units
Ammonia	33	17-80 ug/dL
Thyroid-stimulating hormone	1	0.30 - 5.50 uIU/mL
Vitamin B12	654	211-911 pg/mL
Angiotensin-converting enzyme	23	8-52 U/L
Prothrombin time	13.6	12-14.7 sec
International normalized ratio (INR)	1.07	<4.00
Acetylcholine receptor binding antibody	<0.30	≤0.30 nmol/L
C-ANCA	<1:20	<1:20 titer
P-ANCA	<1:20	<1:20 titer
SSA (Ro) antibodies	2	<20 units
SSB (La) antibodies	3	<20 units
Anti-smooth muscle antibody	Negative	-
Antinuclear antibodies	Negative	-
Lyme disease antibody (IgG)	Negative	-
Lyme disease antibody (IgM)	Negative	-

**Table 3:** Diagnostic Cerebrospinal Fluid Analysis VDRL - venereal disease research laboratory

Component	Value	Reference range & units
Clarity	Clear	Clear
Red blood cell count	0	0 cells/mm^3^
White blood cell count	0	0-5 cells/mm^3^
Glucose	47	40-70 mg/dL
Protein	48	15-45 mg/dL
Lyme disease antibodies	0.17	≤0.99 LIV
VDRL screen	Non-reactive	-
Cryptococcal antigen	Negative	-
Paraneoplastic antibodies	Negative	-
Papanicolaou and Wright-Giemsa cytology	Negative for malignant cells	-

## Discussion

The COVID-19 pandemic prompted an intensive investigation of SARS-CoV-2 and the long-term impact of acute infection. Although neurological manifestations have been documented in previous studies, involvement of the peripheral nervous system is considered an anomaly, reported in only 8.9% (19/214) of cases in a recent study of patients with neurological symptoms preceded by COVID-19 [7]. The pathophysiology of GBS is understood to be the result of molecular mimicry between infectious lipooligosaccharides and human gangliosides, consequently leading to the autoimmune destruction of peripheral nerves [8]. Diagnosis is generally established upon recognition of its characteristic clinical presentation substantiated by findings of albuminocytological dissociation within CSF, and nerve conduction disturbances in electrophysiological studies [2]. Historically, *Campylobacter jejuni*, cytomegalovirus, Epstein-Barr virus, Zika virus, and influenza virus have been recognized as the classic triggers of GBS [9]. In recent years, however, it has also been frequently observed in the setting of vaccinations, surgery, and malignancy [2]. Although there has been an increase in reports of COVID-associated GBS [7], a causal relationship is difficult to establish due to the novelty of SARS-CoV-2 and the changes in social behaviors during the pandemic that have drastically altered the patterns of infectious disease [10].

The clinical spectrum of GBS was established in 2014 by the GBS Classification Group, which consists of the following variants: classic GBS, pharyngeal-cervical-brachial weakness, paraparetic GBS, bifacial weakness with paraesthesias (BFP), Miller Fisher syndrome (MFS), and Bickerstaff brainstem encephalitis (BBE) [6]. In this case report, the patient presented with isolated BFP in an atypical clinical onset of COVID-associated GBS. BFP is characterized by the paralysis of bilateral facial nerves, typically accompanied by peripheral hyporeflexia in the absence of other neurological findings [8]. Recent studies have indicated that this variant in particular may be linked to the COVID-19 vaccine itself [11-13]. In general, BFP has an excellent prognosis and spontaneous symptomatic resolution is expected [8,14]. Due to the lack of therapeutic advantage, treatment is not usually recommended except in critical cases demonstrating rapid progression of lower extremity paralysis or evident risk of cardiorespiratory failure within two weeks of disease onset [15]. The development of significant dysphagia in our patient placed him at substantial risk for respiratory deterioration, which necessitated therapeutic intervention. IVIg and plasma exchange (PE) are most commonly used in the treatment of GBS [15]. Although corticosteroids are considered to be the mainstay of treatment for many immune-mediated conditions, it is proven to be ineffective in GBS [8]. In our case, the patient was responsive to treatment and symptomatic regression was achieved with a five-day course of IVIg.

## Conclusions

GBS encompasses a clinical spectrum of neurological diseases that have been associated with COVID-19. Diagnosis relies on clinical presentation with concordant CSF and electrophysiological findings. As seen in this case report, the postinfectious presentation of bilateral facial paralysis with peripheral hyporeflexia may be the rare manifestation of BFP, a variant of GBS. Treatment with IVIg or PE is typically reserved for critical cases demonstrating rapid progression of lower extremity paralysis or evident risk of cardiorespiratory deterioration within two weeks of disease onset. Otherwise, there is no indication for therapeutic intervention, and the spontaneous resolution of BFP is to be expected.
